# A Novel Approach to Droplet’s 3D Shape Recovery Based on Mask R-CNN and Improved Lambert–Phong Model

**DOI:** 10.3390/mi9090462

**Published:** 2018-09-13

**Authors:** Shizhou Lu, Chenliang Ren, Jiexin Zhang, Qiang Zhai, Wei Liu

**Affiliations:** 1 School of Mechanical, Electrical &Information Engineering, Shandong University at Weihai, Weihai 264209, China; lushizhou@sdu.edu.cn (S.L.); rcl@mail.sdu.edu.cn (C.R.); liuweihpu@mail.sdu.edu.cn (W.L.); 2 School of Mechatronics Engineering, Shanghai Jiao Tong University, Shanghai 201100, China; zhangjiexin@sjtu.edu.cn

**Keywords:** shape from shading, Mask R-CNN, segment highlight region, Lambert–Phong model

## Abstract

Aiming at the demand for extracting the three-dimensional shapes of droplets in microelectronic packaging, life science, and some related fields, as well as the problems of complex calculation and slow running speed of conventional shape from shading (SFS) illumination reflection models, this paper proposes a Lambert–Phong hybrid model algorithm to recover the 3D shapes of micro-droplets based on the mask regions with convolutional neural network features (R-CNN) method to extract the highlight region of the droplet surface. This method fully integrates the advantages of the Lambertian model’s fast running speed and the Phong model’s high accuracy for reconstruction of the highlight region. First, the Mask R-CNN network is used to realize the segmentation of the highlight region of the droplet and obtain its coordinate information. Then, different reflection models are constructed for the different reflection regions of the droplet, and the Taylor expansion and Newton iteration method are used for the reflection model to get the final height of all positions. Finally, a three-dimensional reconstruction experimental platform is built to analyze the accuracy and speed of the algorithm on the synthesized hemisphere image and the actual droplet image. The experimental results show that the proposed algorithm based on mask R-CNN had better precision and shorter running time. Hence, this paper provides a new approach for real-time measurement of 3D droplet shape in the dispensing state.

## 1. Introduction

In the process of microelectronic high-speed dispensing, detecting the 3D shape of droplets online is a necessary precondition for studying the micro-jetting effect and realizing adaptive control of the dispensing process [1,2,3,4]. There is also a huge demand for detecting the 3D shapes of droplets online in many other areas. For example, it is also useful for the droplets formed by microfluidic chips for biological and biomedical applications [5,6,7,8]. 3D shape detection enables the microfluidic chip to control the volume of the droplets more precisely, thereby improving the accuracy of the entire system. However, due to the high viscosity and non-Newtonian behavior of most types of adhesives, the tipping, tailing, and unevenness of the substrate leads to irregular shapes. For the conventional stereo vision method and the structured light approach, problems such as poor real-time performance, low precision, and difficulty in compatibility arise [9,10]. In this context, the use of three-dimensional vision to reconstruct the 3D shape of droplets emerges as a reliable method. The three-dimensional reconstruction method based on monocular vision can be used to derive depth information according to the two-dimensional features of single or multiple images [11]. For example, shape from shading (SFS), proposed by Horn, can be used to reconstruct a three-dimensional model of the object using a single gray image [12]. In comparison with binocular or multi vision, it has the advantages of small calculation, high efficiency, simple operation, and low hardware requirement. Hence, it provides a new approach to solve the problem of the online detection of high-viscosity micro-droplet surface topography. However, for this method, choosing an appropriate illumination reflection model is a problem that must be solved first.

Conventional SFS technology usually adopts a Lambertian model. However, while methods based on the Lambertian model have a high solution speed, the Lambertian model only considers the diffuse reflection component and does not consider the influence of the specular component. Therefore, a large error arises in describing the highlight region, which is not effective for the reconstruction of the 3D shape of droplets. In recent years, some scholars have studied many non-Lambert surfaces for SFS. For example, Phong established a hybrid model which takes specular reflection into account. Although the model has higher recovery accuracy, it runs more slowly [13]. Lee and Kuo suggested a generalized reflectance map model, which is a linear combination of a Lambertian model and a Torrance–Sparrow model for the diffuse reflection and specular reflection [14]. Ahmed and Farag used the Ward model to describe the SFS problem of hybrid surfaces [15]. Oren and Nayar proposed a more accurate Oren–Nayar reflection model based on the Lambertian reflection model, which is closer to the actual reflection characteristics [16]. Based on the Phong model, Breuß proposed the perspective Phong-based SFS (P2SFS) model, which is more in line with the camera's perspective imaging characteristics [17]. Yang replaced the ideal Lambertian model with a radial basis function (RBF) reflection model and used neural networks to solve the problem of nonlinear equations [18]. Wang linearly combined the Oren–Nayar model with the Ward model as the final hybrid reflection model, and used the numerical Hamiltonian and first-order and high-order fixed-point iterative sweeping method to solve the static Hamilton–Jacobi equation [19]. The above-mentioned hybrid illumination models effectively improve the Lambertian model’s low accuracy in the recovery of the specular reflection region. However, these models do not effectively deal with the difference between the diffuse reflection component and the specular reflection component in different regions. Besides, using the same algorithm to reconstruct the shape may lead to complexity of the calculation and slow convergence rate. Therefore, from the perspective of combinatorial optimization, combining the characteristics of each algorithm, this paper proposes a Lambert–Phong combination model that uses the Lambertian model and the Phong model to reconstruct the shape of the diffuse and highlight regions, respectively. The first step of this method is to identify the highlight regions.

In recent years, the popular highlight detection methods mainly include analysis based on chromaticity or polarization analysis of light. For example, Wolff used the difference between specular and diffuse reflection characteristics for highlight region detection [20]. Nayar used color information and polarization information to estimate highlight regions. However, many of these methods cannot be applied to the SFS algorithm [21]. In addition, Alsalsh applied a hybrid color attributes and wavelet-based edge projection approach to accurately identify the affected regions [22]. However, the detection of the highlight region is still incomplete, and reconstructing highlight region’s 3D shape still lacks accuracy.

Recently, more and more researchers have applied improved convolutional neural networks (CNNs) to classify and predict objects at the pixel level [23,24,25]. From fully convolutional networks (FCNs), Fast R-CNN (regions with convolutional neural network features), to Faster R-CNN [26,27,28], the running time and accuracy have been continuously improved. However, they are yet to be further improved. In this context, the latest Mask R-CNN combines the existing Faster R-CNN and FCN technologies to add a parallel branch for predicting the target mask on its existing branch for border recognition [29]. It greatly improves the accuracy of recognition and classification at pixel-level.

**Figure 1:**
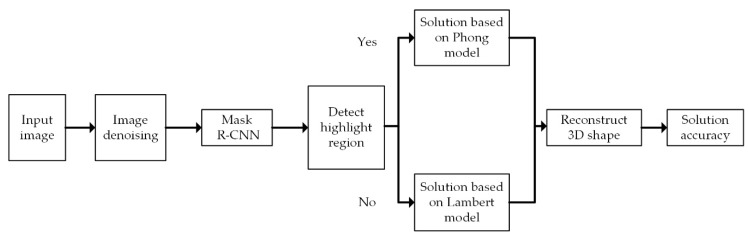
Algorithm flow chart.

Therefore, according to the demand of 3D shape recovery of droplets in the field of microelectronic packaging, the Mask R-CNN method is applied for highlight region segmentation in this paper by considering the problem of slow speed in calculation in the conventional SFS hybrid reflection method. The Lambert–Phong hybrid reflection model is proposed, combining the advantages of the fast solution speed of the Lambertian method and the high accuracy of the Phong method at the highlight region. First, the algorithm denoises the input droplets image. Then, the Mask R-CNN depth learning neural network is used to perform highlight area segmentation, so as to obtain the highlight area coordinates for the next step. Next, the optimization algorithm is linearized to the highlight region and non-highlight region at the pixel level. Then, the two parts are combined to realize the 3D shape reconstruction of the droplet. Finally, the precision and running speed of the composite image and the real image are analyzed, respectively. The algorithm flow is shown in Figure 1.

## 2. Highlight Region Segmentation Based on Mask R-CNN

**Figure 2:**
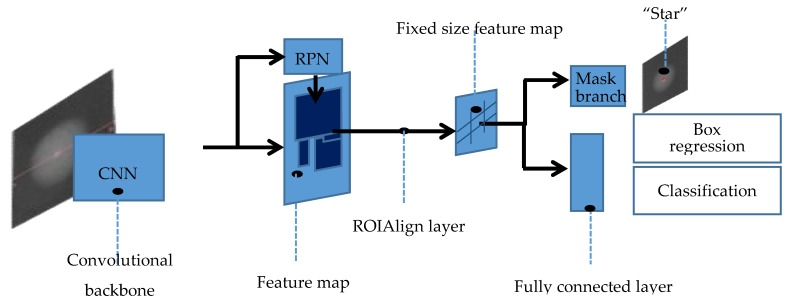
Mask R-CNN flow chart. CNN: convolutional neural network; ROI: region of interest; RPN: region proposal network.

Mask R-CNN is a conceptually simple and flexible method for object instance segmentation that uses the same first half portion of the program as Faster R-CNN: a region proposal network (RPN) is utilized for region of interest extraction [30]. While predicting the box offset and class for each region of interest, it outputs a binary mask. This method does not require a compression operation as Faster R-CNN does. Note that the FCN can be applied to each region of interest (ROI) for the prediction of a segmentation mask, since the mask directly represents the correspondence between pixels by convolution. Figure 2 shows the specific flow of this method:

- Input of the normalized image into the main network
  To facilitate the generation of the mask, the fixed 512 × 512 images are input into the network [31], which have undergone median filtering and normalization.
- Feature extraction and generation of regions of interest
  The image is sent to the main network to extract the data, and then the region proposal network is used to find the region of interest. Subsequently, a layer called ROIAlign is adopted that accurately aligns the extracted features with the input to improve the accuracy of the object mask.
- Proposing the box offset, the class, and the mask
  A *n* × *n* sliding window is used to generate a one-dimensional fully connected feature in the fifth convolutional layer of the network. Ultimately there are three branches generated [32], which contain the information to predict: reg-layer, cls-layer, and object mask. Thence, the first two branches are used for bounding-box classification and regression in parallel. The third branch is used to output the binary mask of the highlight feature called “Star”.

**Figure 3:**
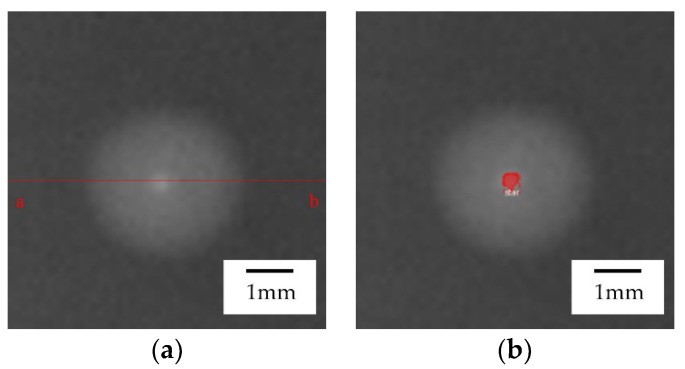
(**a**) Binary droplet image of feature to be extracted. (**b**) Experimental image after detection by Mask R-CNN (where the red area is the highlight area and the feature area is called “star”).

With completion of the Mask R-CNN training, the test image is input into the network, and then it is feasible to obtain and output the highlight position information. Finally, the diffuse reflection area and the highlight area of the droplet to be tested can be spotted. Clearly, the red area in Figure 3 is the highlight area identified by the experiment.

## 3. 3D Shape Recovery Based on Combined Optimization Model

In the case of an ideal diffuse reflector, the effects of diffuse reflection components are idealistically considered. The reflection model is as follows [33]:

(1) $$E_{l}\left(x,y\right)=R_{l}(p,q)=\rho_{l}\frac{pp_{m}+qq_{m}+1}{\sqrt{p^{2}+q^{2}+1}\sqrt{p_{m}^{2}+q_{m}^{2}+1}},$$

where (x,y) is the position of the corresponding pixel, (p,q) is the gradient information of the image pixels, R(p,q) is the Lambertian model reflection function, $E_{l}\left(x,y\right)$ is the luminance information of the image after normalization, and $\rho_{l}$ is the surface reflection coefficient of the diffuse reflection component.

In fact, most object surface reflections can be considered as a linear combination of diffuse and specular components, since they both exist on the surface of the object. This relationship is precisely described by the Phong model:

(2) $$Ep\left(x,y\right)=Rp\left(p,q\right)=(1-w)\rho_{l}\frac{pp_{m}+qq_{m}+1}{\sqrt{p^{2}+q^{2}+1}\sqrt{p_{m}^{2}+q_{m}^{2}+1}}+w\rho_{s}{\left(\frac{pp_{h}+qq_{h}+1}{\sqrt{p^{2}+q^{2}+1}\sqrt{p_{h}^{2}+q_{h}^{2}+1}}\right)}^{n_{s}},$$

where $\rho_{l}$ and $\rho_{s}$ are the surface reflection coefficients of the diffuse and specular components, respectively, w∈[0,1] is the smoothing factor, $n_{s}$ is the specular reflection factor, $\left(p_{m},q_{m},-1\right)$ is the direction vector of the light source, and $\left(p_{k},q_{k},-1\right)$ is the source direction vector—the direction vector on the intersection of the light source and the camera.

It is obvious that the equation adds the specular component compared with the Lambertian reflection model. Then, we combine the two models and propose $k_{h}$ in the model as a highlight factor to obtain an optimized model:

(3) $$\begin{array}{l} E\left(x,y\right)=R\left(p,q\right)=(1-k_{h})\frac{pp_{m}+qq_{m}+1}{\sqrt{p^{2}+q^{2}+1}\sqrt{p_{m}^{2}+q_{m}^{2}+1}}+k_{h}(1-w)\rho_{l}\frac{pp_{m}+qq_{m}+1}{\sqrt{p^{2}+q^{2}+1}\sqrt{p_{m}^{2}+q_{m}^{2}+1}}+\\ k_{h}w\rho_{s}{\left(\frac{pp_{h}+qq_{h}+1}{\sqrt{p^{2}+q^{2}+1}\sqrt{p_{h}^{2}+q_{h}^{2}+1}}\right)}^{n_{s}} \end{array}$$

where $k_{h}=1$ corresponds to the highlight region, and then Phong model is used. $k_{h}=0$ corresponds to the diffuse reflection region, and then the Lambertian reflection model is used.

Therefore, Equation (3) is used to calculate and identify different regions of the Lambert–Phong hybrid model. It is applicable to solve the Lambertian model by using the linearization method in the diffuse reflection region:

(4) $$f(z_{i,j})=E_{i,j}-R(z_{i,j}-z_{i,j-1},z_{i,j}-z_{i-1,j})=0\;$$

Then, Taylor expansion is applied:

(5) $$f(z_{i,j})\approx f(z_{i,j}^{n-1})+(z_{i,j}-z_{i,j}^{n-1})\frac{df}{dz_{i,j}}(z_{{}_{i,j}}^{n})\;$$

Therefore, the following equation can be obtained:

(6) $$z\begin{array}{c} n\\ i,j \end{array}=z\begin{array}{c} n-1\\ i,j \end{array}-\frac{f(z\begin{array}{c} n-1\\ i,j \end{array})}{\frac{df}{dz_{i,j}}(z\begin{array}{c} n-1\\ i,j \end{array})}.$$

Therefore, when given the initial value, iterative calculation can be performed to obtain the final iteration result called $z_{l}=z\begin{array}{c} n\\ i,j \end{array}$, which is the height of each point in the images. Similarly, the Phong hybrid model is used to solve the highlight region.

Because E(x,y)=R(p,q), $E_{x}=R_{x}$, $E_{y}=R_{y}$, the original equation is corrected by introducing the image gradient weighting coefficient: λ∈[0,1].

Then, the following equation is obtained:

(7) $$E\left(x,y\right)+\lambda \left(E_{x}\left(x,y\right)+E_{y}\left(x,y\right)\right)=R\left(p,q\right)+\lambda \left(R_{x}\left(p,q\right)+R_{y}\left(p,q\right)\right),$$

where

(8) $$\begin{array}{l} E_{x}=\frac{\partial E}{\partial x}=\frac{\partial R}{\partial x}=\frac{\partial R}{\partial p}\frac{\partial p}{\partial x}+\frac{\partial R}{\partial q}\frac{\partial q}{\partial x}\\ E_{y}=\frac{\partial E}{\partial y}=\frac{\partial R}{\partial y}=\frac{\partial R}{\partial p}\frac{\partial p}{\partial y}+\frac{\partial R}{\partial q}\frac{\partial q}{\partial y} \end{array}$$

A new expression of the objective function can be obtained based on the central difference of the discretization of the target equation.

(9) $$\begin{array}{l} F(p,q)=f(E,Ex,Ey)-f(R,Rx,Ry)\\ \approx F(z_{i,j},z_{i\pm 1,j},z_{i,j\pm 1},z_{i\pm 1,j\pm 1},z_{i\pm 2,j\pm 1},z_{i\pm 1,j\pm 2})\\ =0 \end{array}$$

With the acquisition of this equation, the Newton iteration method is used to solve the value of the height *z*, and its equation structure is similar to the Lambertian linear method.

Finally, the following equation is established:

(10) $$z\begin{array}{c} n+1\\ i,j \end{array}=z\begin{array}{c} n\\ i,j \end{array}\;—\sum_{(i,j)\in S}\beta_{i,j}\frac{F_{k}(Z)}{\frac{dF_{k}(Z)}{dz_{i,j}}}|{}_{z=z^{n}},$$

where $\beta_{i,j}$ is the harmonic coefficient of the pixel, which is generally set as 1/13. With the completion of the Newton iteration, the convergence value $z_{p}=z\begin{array}{c} n+1\\ i,j \end{array}$ is obtained, which is the final height of the 3D contour solved based on the Phong hybrid reflection model.

Finally, the height value solved by the optimization algorithm of this paper is obtained by combining the height values obtained by the two models:

(11) $$Z_{a}=(1-k_{h})\times z_{l}+k_{h}\times z_{p}.$$

## 4. Three-Dimensional Shape Recovery Based on Combined Optimization Model

**Figure 4:**
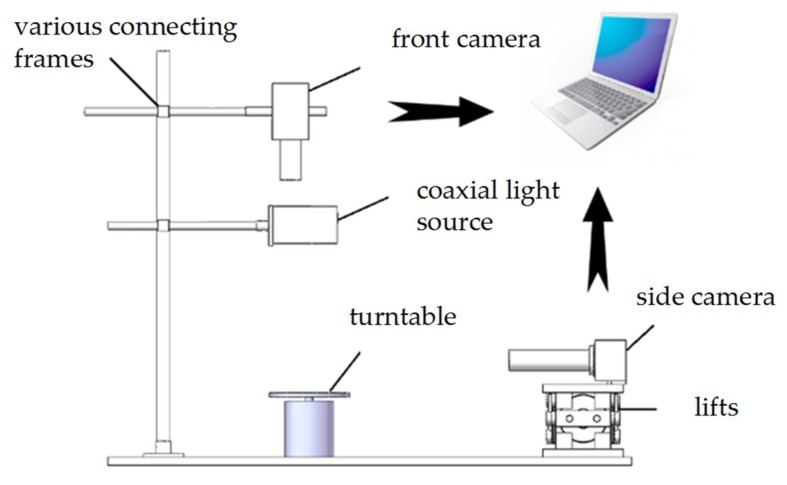
Schematic diagram of experimental equipment.

As shown in Figure 4, the droplet image acquisition experimental platform designed in this paper was mainly composed of a front camera, a coaxial light source, a turntable, and a side camera for precision calibration, power supply, computer, and various connecting frames.

In order to facilitate the experiment, the droplet was placed on the center of the turntable, and the positions of the light source and the camera were adjusted to make it perpendicular to the turntable. Meanwhile the position of the side camera was adjusted to make it parallel to the turntable. The side camera was used to collect the positive side image of the droplet, and it was assumed to be the theoretical height data for the side view of the droplet. The theoretical height data of the side view of the droplet were used for comparison with the height data reconstructed by the algorithm and as the final experimental data accuracy evaluation standard.

### 4.1. Precision Analysis of Synthetic Image

**Figure 5:**
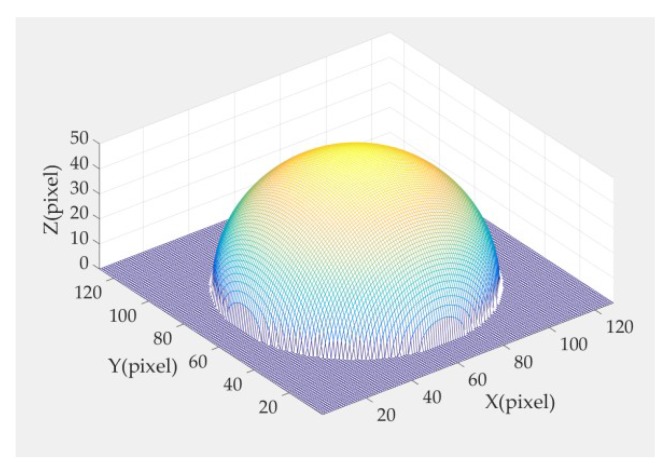
Composite hemisphere.

It is difficult to detect the 3D shape of a real image. Therefore, the composite image was first to be tested. In this section, the composite hemisphere is used for 3D reconstruction. The equation for the hemisphere is as follows:

(12) $$z(x,y)={\left(r^{2}-{\left(x-x_{o}\right)}^{2}-{\left(y-y_{o}\right)}^{2}\right)}^{1/2},$$

where $\left(x_{o},y_{o},-1\right)$ is the position coordinate of the center of the ball, *z* is the height of the corresponding position (x,y), and *r* is the radius. Here, we assign *r* = 50 pix. The composite hemisphere is shown in Figure 5.

**Figure 6:**
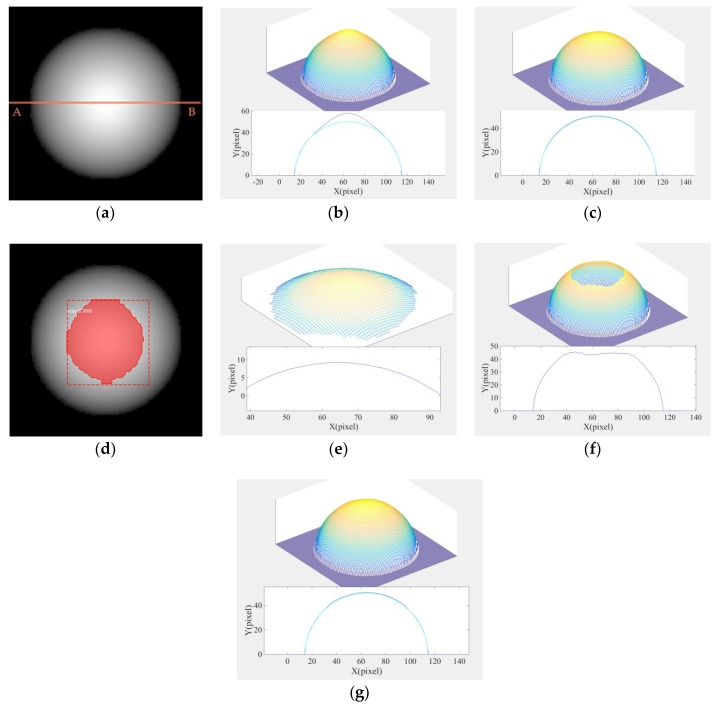
(**a**) Original grayscale composite image (A–B is the cut line for the cross section); (**b**) 3D shape of the composite image and the side cross section contrast diagram using the Lambertian model algorithm (dark blue is the cross section of reconstruction, light blue is the cross section of the real shape); (**c**) 3D shape of the composite image and the side cross section contrast diagram using the Phong hybrid model algorithm; (**d**) highlight detection’s results of the composite image; (**e**) 3D shape of the composite image at the highlight region using the Phong hybrid model algorithm; (**f**) 3D shape of the composite image at the non-highlight region using the Lambertian model algorithm; (**g**) 3D shape of the composite image and the side cross section contrast diagram using the Lambert–Phong model algorithm.

Figure 6a is a grayscale image corresponding to 14.3% high gloss. The parameters were as follows: $n_{s}=10$, *w* = 0.5, $k_{h}=1$, $p_{l}=0.857$, $p_{s}=0.143$. The direction of the light source $\left(p_{m},q_{m},-1\right)$ was (0, 0, −1).

As shown in Figure 6b, the Lambertian reflection model was sensitive to highlight regions—even in the composite map there was a distortion district. However, the reconstruction effect was better in the diffuse reflection region. Figure 6c illustrates that the linearization of the Phong model was effective, and therefore could effectively deal with the highlight regions. However, it consumed too much time. Therefore, in this paper, based on the combination optimization, Mask R-CNN was used to segment the highlight region of the composite image as shown in Figure 6d. The coordinate information after segmentation can be used by the 3D shape reconstruction algorithm to improve the accuracy of reconstruction.

We solved the linearization of the Phong model for the highlight region and the linearization of the Lambertian model for the non-highlight region, respectively. The results of the three-dimensional reconstruction are shown in Figure 6e,f. We combined these two results to bring out the final experimental data of the algorithm.

Figure 6g shows the best solution, as there was no distortion in both highlight and non-highlight regions, and the calculation speed was relatively faster. In this paper, the maximum cross-sectional height average relative error and height root mean square error of the composite image were solved respectively. The formulas are as follows:

(13) $$ARE=\frac{1}{n}\sum_{1}^{n}\left(\sum_{x}(Z_{a}-z)/z)/size(x\right),$$

(14) $$RMSE=\frac{1}{n}\sum_{1}^{n}\sqrt{\frac{1}{m}{\sum_{x=1}^{m}\left(z\left(x\right)-Z_{a}\left(x\right)\right)}^{2}},$$

where *ARE* is the height average relative error, *RMSE* is the height root mean square error, *n* is the total number of processed images, *m* is the total number of pixels of the largest cross section of the composite sphere, $Z_{a}$ is the height value after the reconstruction, and *z* is the actual height value. The running time and accuracy are shown in Table 1.

Table 1 indicates that the Lambert–Phong model had the smallest *ARE* value among the three compared models, and a slightly larger *RMSE* value than the Phong model. The CPU time was only 0.73761 s, which is much shorter than the Phong model. Thus, we conclude that the proposed Lambert–Phong model inherits the advantages of high efficiency from the Lambertian model and high accuracy from the Phong model.

From the above experimental data, there was a large error using the diffuse reflection model in the highlight region because it ignores the specular reflection components of the highlight region, whereas the Lambert–Phong optimization model developed in this study solves the distortion and time problems of the two algorithms, and the reconstruction effect was better than that of the single algorithm.

### 4.2. Precision Analysis of Real Image

In this section, the above algorithm is applied to the 3D shape reconstruction experiment of the droplets, and the experimental results are compared with different algorithms. Since the 3D shape of the real tiny droplets is difficult to detect accurately, this paper calculates the accuracy through the maximum external contour of the side image.

**Figure 7:**
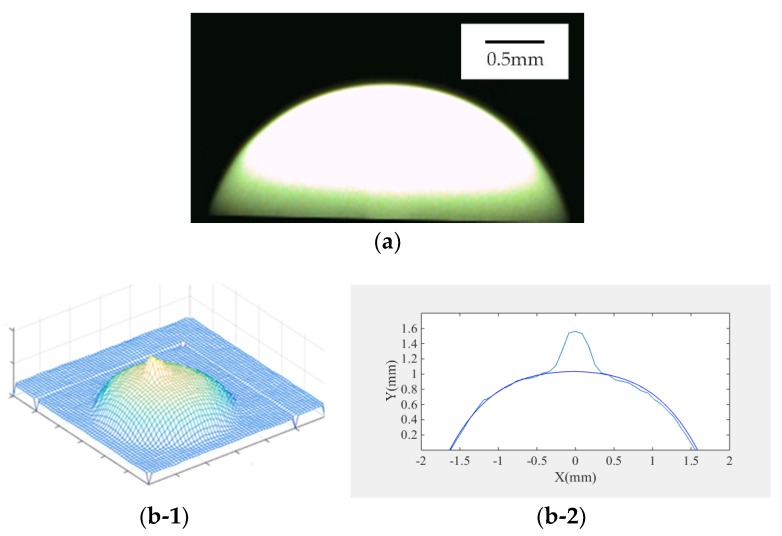

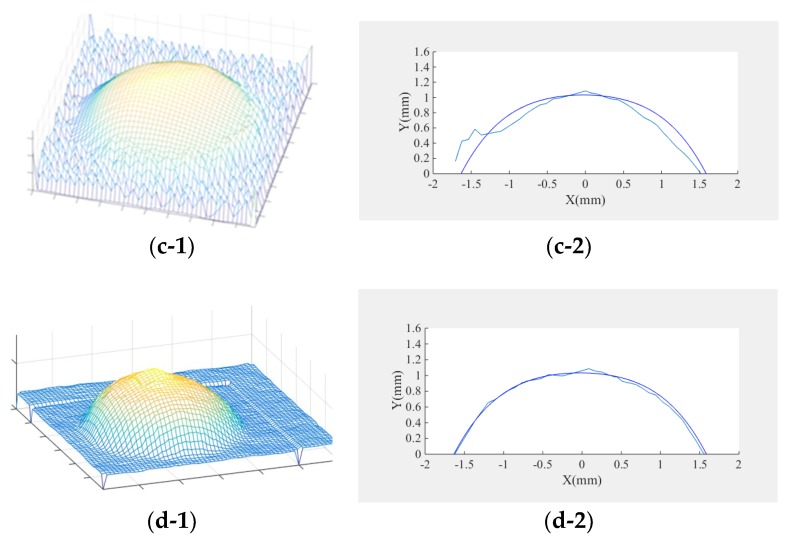
(**a**) Real profile of the droplet; (**b-1**) 3D shape reconstruction of the real image using the Lambertian model algorithm; **(b-2**) 3D shape of the real image and the side cross section contrast diagram using the Lambertian model algorithm; (**c-1**) 3D shape reconstruction of the real image using the Phong hybrid model algorithm; (**c-2**) 3D shape of the real image and the side cross section contrast diagram using the Phong hybrid model algorithm; (**d-1**) 3D shape reconstruction of the real image using the Lambert–Phong model algorithm; (**d-2**) 3D shape of the real image and the side cross section contrast diagram using the Lambert–Phong model algorithm.

The experimental results of the 3D shape in Figure 7b demonstrate that the diffuse reflection model solved by linearization could not accurately describe the specular component of the surface of the object. Therefore, a large distortion occurred in the highlight region, which affected the accuracy of reconstruction greatly. Comparison with the experimental results of the Phong hybrid model shown in Figure 6c and Figure 7c demonstrates that although the reconstruction effect of the highlight region and the non-highlight region was better in the composite image, the distortion phenomenon appeared in the droplet’s boundary in the real image, which reduced the accuracy to a certain extent. The results of the combined Lambert–Phong optimization model proposed in this paper are shown in Figure 7d. It is clear that the experimental result was more accurate, the reflection characteristics of the droplet surface could be expressed more accurately, and the reconstruction error was also well-solved. Hence, it can be concluded that the Lambert–Phong model was applicable for the droplet in this case.

In this section, the data of the three algorithms are compared and the maximum cross-sectional height average relative error and height root mean square error are calculated. As shown in Figure 3a, the cross section of the real image was taken with a–b as the cross-sectional line. The cross section contrast diagrams of the three algorithms are shown in Figure 7b-2, Figure 7c-2, and Figure 7d-2. Based on the above-mentioned formulas, the error values of the three models are listed in Table 2. It can be seen from the error table that the accuracy of the algorithm proposed in this paper was higher than that of the diffuse reflection model and Phong hybrid model, and therefore it is suitable for the three-dimensional reconstruction of industrial droplets. In the real image, the solution speed of this model was still faster than other models, so the model is applicable for the droplets’ experimental environment.

## 5. Conclusions and Prospect

In this paper, a novel approach based on Mask R-CNN and improved Lambert–Phong model is carried out to reconstruct the micro-droplet’s 3D shape with the advantages of high accuracy and efficiency. Firstly, Mask R-CNN is used to segment the highlight region and diffuse reflectance region of the droplets, and then the Lambert and Phong models are combined to reconstruct the 3D shapes of diffuse reflectance region and highlight region, respectively. Finally, the above two results are combined to get the final 3D shape of the droplets. In the experiment, the reconstruction errors of 3.81% and 8.06% in the composite image and the actual droplet image based on Lambert–Phong model proposed in this paper were both smaller than the other algorithms based on a single model, which shows that the algorithm in this paper had good experimental precision and running speed. This study provides an effective way for monitoring the volume and shape of droplets in the microelectronic dispensing area in real-time. In the future, studies about the 3D shape recovery of some irregularly shaped droplets and a droplet jetting volume control method based on the proposed shape recovery algorithm will be explored deeply.

## Figures and Tables

**Table 1:** Comparison of height average relative error (*ARE*), height root mean square error (*RMSE*), and operational speed of the three algorithms for a composite image.

Method	*ARE* (%)	*RMSE* (pix)	CPU Time (s)
Lambertian model	5.66	3.983	0.13396
Phong model	3.95	0.403	7.44460
Lambert–Phong model	3.81	0.475	0.73761

**Table 2:** Comparison of height average relative error, height root mean square error, and operational speed of three algorithms for the real image.

Method	*ARE* (%)	*RMSE* (pix)	CPU Time (s)
Lambertian model	8.99	0.162	0.00089
Phong model	17.00	0.145	0.14126
Lambert–Phong model	8.06	0.032	0.01525
